# An Individually Optimized Protocol of Contrast Medium Injection in Enhanced CT Scan for Liver Imaging

**DOI:** 10.1155/2017/7350429

**Published:** 2017-07-10

**Authors:** Shi-Ting Feng, Hongzhang Zhu, Zhenpeng Peng, Li Huang, Zhi Dong, Ling Xu, Kun Huang, Xufeng Yang, Zhi Lin, Zi-Ping Li

**Affiliations:** ^1^Department of Radiology, The First Affiliated Hospital, Sun Yat-sen University, 58th, The Second Zhongshan Road, Guangzhou, Guangdong 510080, China; ^2^Faculty of Medicine and Dentistry, University of Western Australia, Perth, WA, Australia

## Abstract

**Objective:**

To investigate the effectiveness of a new individualized contrast medium injection protocol for enhanced liver CT scan.

**Methods:**

324 patients who underwent plain and dual phase enhanced liver CT were randomly assigned to 2 groups: G1 (*n* = 224, individualized contrast medium injection protocol); G2 (*n* = 100, standard contrast medium injection with a dose of 1.5 ml/kg). CT values and ΔHU (CT values difference between plain and enhanced CT) of liver parenchyma and tumor-liver contrast (TLC) during hepatic arterial phase (HAP) and portal venous phase (PVP) and contrast medium dose were measured. The tumor conspicuity of hepatocellular carcinoma (HCC) between two groups was independently evaluated by two radiologists.

**Results:**

The mean contrast medium dose of G1 was statistically lower than that of G2. There were no significantly statistical differences in CT values and ΔHU of liver parenchyma during HAP, TLC values during HAP, and PVP between two groups. The CT values and ΔHU of liver parenchyma during PVP of G2 were significantly higher than those of G1. Two independent radiologists were both in substantial conformity in grading tumor conspicuity.

**Conclusion:**

Using the individually optimized injection protocol might reduce contrast medium dose without impacting on the imaging quality in enhanced liver CT.

## 1. Introduction

The use of contrast enhanced computed tomography (CT) with iodinated contrast medium (ICM) has significantly improved the accuracy of imaging diagnosis. The rapid development of CT technologies has led to an increase in world-wide usage of ICM. This also results in an increase in its associated adverse reactions, where contrast-induced nephropathy (CIN) is one of the most concerning adverse effects by far. As early as 2001, M. M. Waybill and P. N. Waybill [1] reported that CIN had become the third leading cause of all hospital-acquired renal insufficiency. Since kidney is the primary organ where ICM is metabolized, higher dose of ICM may cause greater damage to the kidney, hence resulting in higher incidence of CIN [2]. Davidson et al. [3] reported that incidence of CIN proportionally correlates with the contrast medium dose used especially amongst high-risk populations with preexisting renal insufficiency or diabetic neuropathy.

Therefore, on the premise of ensuring the quality and display capability of CT images, reasonable reduction in contrast medium dose may effectively prevent and reduce the incidence of adverse effects associated with enhanced CT scans. Various methods had been previously proposed to reduce the contrast medium dose, including individualized weight-based protocols [4–8], adjustment on the injection time or flow rate of contrast administration [9–11], and the use of additional saline flush [12–14]. Out of the various options, previous reports had demonstrated that personalized weight-based contrast medium injection protocol is an ideal method to reasonably reduce the injection dose of contrast medium [8].

Personalized patient protocol technology abdomen module is a new intelligent platform, which enables the generation of individualized contrast medium injection protocol based on patient characteristics (such as weight), contrast medium properties (such as iodine content), and other procedure parameters (such as scan timing). P3T™ (Bayer Healthcare, Berlin, Germany) is designed as an individualized contrast medium injection protocol software adapting the iodine delivery rate and total iodine load based upon a nonlinear relationship between patient weight and scan duration in order to achieve diagnostic attenuation. By using patient weight, scan duration, contrast medium concentration, and timing attributes of a test bolus scan, P3T facilitates customizing injection protocol for each patient and procedure. Previous studies have shown that this customized injection software could lead to diagnostic and comparable attenuation values in the coronary CTA for every individual patient and a more efficient use of contrast medium dose [15, 16]. However, the application of this individually optimized protocol of contrast medium injection in liver imaging has not been evaluated previously.

In this study, we aimed to evaluate whether this new contrast medium injection protocol can reduce the contrast medium dose used in enhanced CT scan for liver imaging without limiting the quality of the images.

## 2. Materials and Methods

### 2.1. Patients

This prospective study was conducted in accordance with ethical guidelines for human research and was compliant with the Health Insurance Portability and Accountability Act (HIPAA). The study has been approved by the Institutional Review Board (IRB) or ethical committee. Written informed consent was obtained from all patients in the study.

All patients who underwent liver CT scan in our hospital between January 2013 and December 2015 were included in this study. Exclusion criteria were large liver lesions (diameter > 5 cm), diffuse liver diseases such as cirrhosis (suggestive CT findings include abnormal size and shape of liver and spleen, inhomogeneous liver appearance with regenerating nodules and/or signs of portal vein hypertension [17]) and multiple metastases, postliver resection, severe fatty liver (liver density lower than spleen in unenhanced CT), cardiac insufficiency (Grades II, III, and IV, NYHA), liver insufficiency (liver function Child-Pugh B and C), renal failure (1–5 stages, chronic kidney disease (CKD)), and known allergies to contrast medium. In the end, a total of 324 cases were included.

All patients were randomly assigned to either Group 1 (G1) or Group 2 (G2). 224 patients were randomized into G1, with mean age of 47.7 ± 11.7 years and mean weight of 59.8 ± 10.9 kg; 100 patients were randomized into G2, with mean age of 53.9 ± 12.0 years and mean weight of 61.8 ± 10.4 kg. There were no statistical differences in patient age and weight between G1 and G2 (*P* > 0.05). A total of 38 patients with histopathologically proven hepatocellular carcinoma (HCC) were included in the study. 23 patients (18 male and 5 female; mean age of 63.4 years) were randomized to G1 and 15 patients (13 male and 2 female; mean age of 58.3 years) to G2.

### 2.2. CT Scan Protocols

All patients were scanned using a 64-detector row CT machine (Aquilion 64, Toshiba Medical System, Tokyo, Japan) using same scanning parameters as follows: tube voltage, 120 kV; tube current, 250 mAs; rotation time, 0.358 s; field of view, 400 mm; reconstruction interval, 1 mm; slice thickness, 0.8 mm. All patients underwent both unenhanced and enhanced CT scans during hepatic arterial phase (HAP) and portal venous phase (PVP). According to Mihl et al. and Tu et al. [16, 17], all the enhanced CT scans during HAP and PVP in the present study started at 35 s and 65 s, respectively, after the contrast injection, from the level of diaphragm to inferior hepatic edge. Both groups received the same contrast medium with an iopromide concentration of 300 mgI/mL (Ultravist, Bayer, Germany) injected at a flow rate of 3 mL/s. G1 adopted an individually optimized protocol (P3T abdomen module, Medrad Inc.) of the platform, which automatically calculates the contrast medium dose based on the weight of each patient by using weight factor dosing method calculated from the following formula:

(1) Contrast  volume  ml=Weight  Factor  gI/kg∗patient  Weight  kgContrast  Concentration  mg/ml∗1000.

The weight factor is expressed in grams of iodine per kilogram of patient weight and specified as 0.4 gI/kg. Contrast medium concentration is 300 mgI/kg. The formula uses both patient weight and contrast concentration for determining an individualized contrast dose. This module automates the calculation of individualized contrast injection protocols. By providing the patients' weight, iodine concentration, and either the flow rate or duration for the contrast injection protocol, P3T Abdomen will generate a protocol specifically tailor to the patient by delivering customized contrast through weight-based calculation.

According to Megibow et al. [7], acceptable image quality can be obtained for most patients by using low osmolar contrast medium with an iodine concentration of 300 mg/ml given at a dose of 1.5 ml/kg based on body weight. Therefore, in this study, G2 candidates received a standard contrast medium injection protocol with a contrast medium to weight dose of 1.5 ml/kg.

### 2.3. Quantitative Image Analysis

Quantitative analysis was later performed on the workstation (HP Workstation XW8200, Vitrea 2, Version 3.7). CT values of unenhanced liver parenchyma, CT values of liver parenchyma during HAP and PVP, and CT values of the portal vein during PVP were measured via regions of interest (ROIs) on the axial images. The CT values of liver parenchyma were measured in three liver sections (right anterior, right posterior, and left lateral segments) and the mean values were calculated. The ROI was circular with a fixed area of 0.5 cm^2^. Caution was taken during measurement to avoid the interference of vessels, edges, bile duct, intestine, and so on ROI was placed at the portal vein trunk, and the edges of ROI should be as close as possible to the edge of the vessel wall on both sides of the portal veins. The liver parenchyma enhancement ΔHU during HAP and PVP was defined as the difference in CT values of liver parenchyma during HAP and PVP compared to unenhanced CT values, respectively.

Tumor-liver contrast (TLC) was used to represent the tumor conspicuity of lesions during HAP and PVP. TLC was previously defined by Baron [18] as the conspicuity of a hepatic tumor expressed by the attenuation difference between the tumor and the hepatic parenchyma. According to Yanaga et al. [19], an attempt was made to maintain a constant ROI area of approximately 2 cm^2^ within the range of 0.8–2.0 cm^2^. In the patients with less than three lesions, the mean TLC values were obtained and calculated from all the lesions; in patients with three or more lesions, the mean TLC values was obtained from the average of the three largest lesions.

### 2.4. Qualitative Image Analysis

CT examinations were performed in both G1 and G2 patients which contained 23 and 15 cases, respectively, of histopathologically proven HCC. The cases were randomly evaluated by two radiologists independently, both with a minimum experience of 15 years specializing in abdominal imaging, both blinded to the clinical data. A three-level grading system was utilized for evaluation: Grade 1, poor (tumor barely shown); Grade 2, fair (tumor is shown but not as clear as Grade 3); and Grade 3, excellent (tumor clearly shown and presence of tumor can be described with confidence). Each case was reviewed independently and image quality grading was assigned accordingly by the consensus of the two radiologists [19].

### 2.5. Statistical Analysis

Analysis was performed using SPSS (SPSS, Version 13.0, Chicago, IL, USA). The contrast medium dose, the CT values of liver parenchyma during HAP and PVP, the CT values of portal vein during PVP, TLC values, and liver parenchyma ΔHU during HAP and PVP in G1 and G2 were presented as mean ± standard deviation (SD). These values were further analyzed by two independent samples *t*-test or Wilcoxon rank sum test, depending on the adherence to normal distribution. If statistically significant differences were observed in the contrast medium dose between G1 and G2, the patients in both groups would be further divided into three subgroups based on patient body weight (≦50 kg, >50 kg and <65 kg, and ≧65 kg) where the mean values were further compared between G1 and G2 corresponding subgroups. The Pearson product-moment correlation coefficient or Spearman rank correlation, depending on the adherence to normal distribution, was used to assess whether linear correlation can be extracted between the contrast medium dose and the liver parenchyma during HAP and PVP and between CT values of the portal vein during PVP between the two groups.

The conformity assessment of visual grade by the two radiologists was subsequently evaluated for interobserver variability using kappa test. The scale of conformity for interobserver agreement according to kappa coefficient was as follows: less than 0.20, poor; 0.21–0.40, fair; 0.41–0.60, moderate; 0.61–0.80, substantial; and 0.81–1.00, almost perfect [20].

## 3. Results

Normality test showed that the distribution of these data was all skew in G1 and G2, including CT values of liver parenchyma during HAP and PVP, CT values of portal vein during PVP, liver parenchyma ΔHU during HAP and PVP, and contrast medium dose. Thus, Spearman correlation test was used to compare the differences between the two groups.

**Figure 1:**
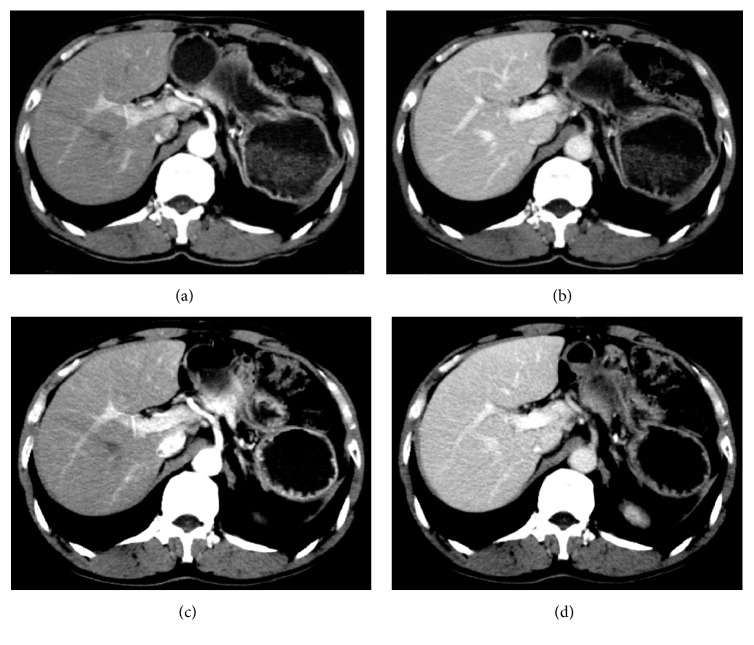
The follow-up CT image pairs of a 63-year-old male patient with a body weight of 47 kg after insulin tumor resection. Images (a) and (b) were scanned with injection protocol in G1 (contrast medium dose: 78 ml). The liver parenchyma CT values during HAP and PVP, portal vein CT value during PVP, and liver parenchyma ΔHU during HAP and PVP were 75 HU, 106 UH, 136 HU, 35 HU, and 66 HU, respectively. Images (c) and (d) were scanned with injection protocol in G2 (contrast medium dose: 87 ml). The liver parenchyma CT values during HAP and PVP, portal vein CT value during PVP, and liver parenchyma ΔHU during HAP and PVP were 77 HU, 115 HU, 153 HU, 37 HU, and 75 HU, respectively. The liver anatomical structures of the two pairs of images were both visualized clearly.

The anatomical structure of liver (liver parenchyma, blood vessels, etc.) was clearly displayed in G1. No obvious difference was observed in the anatomical structure of liver during HAP and PVP between G1 and G2 through initial visual assessment (Figure 1).

The quantitative measurement of CT values of liver parenchyma during HAP and PVP, CT values of portal vein during PVP, liver parenchyma ΔHU during HAP and PVP, and the contrast medium dose is shown in Table 1. There were no statistical differences in the CT values of liver parenchyma and liver parenchyma ΔHU during HAP between the two groups. However, there were statistically significant differences in CT values of liver parenchyma during PVP, CT values of portal vein during PVP, liver parenchyma ΔHU during PVP, and contrast medium dose required between the two groups (Table 1). The contrast medium dose used in G1 was reduced by an average of 14.8 ml when compared to G2.

**Figure 2:**
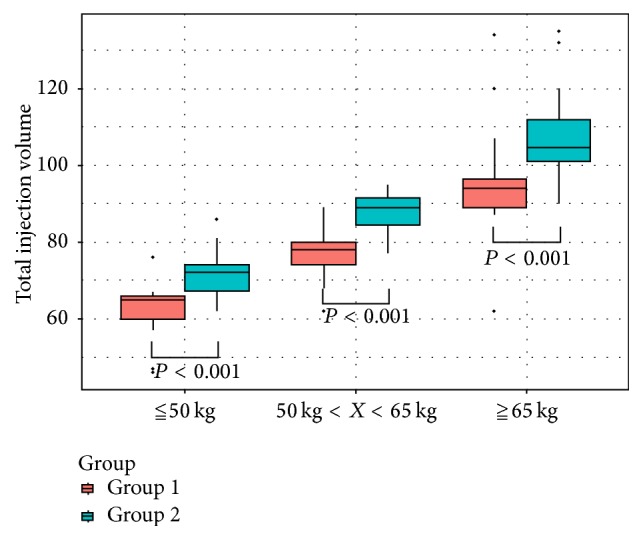
The mean contrast medium dose used in the three weight-based subgroups (≦50 kg, >50 kg and <65 kg, and ≧65 kg) were 62.87, 77.17, and 94.05 ml, respectively, in G1 and 71.38, 88.03, and 106.92 ml, respectively, in G2. There was statistical difference between the subgroups of the same weight range in G1 and G2.

The mean contrast medium doses used in the three weight-based subgroups (≦50 kg, >50 kg and <65 kg, and ≧65 kg) were 62.87, 77.17, and 94.05 ml, respectively, for G1, and 71.38, 88.03, and 106.92 ml, respectively, for G2 (Figure 2). Statistical significant differences were observed between the corresponding subgroups of the same weight range in G1 and G2. The contrast medium doses of the three subgroups in G1 were reduced by 8.51, 10.86, and 11.95 ml, respectively, when compared to the corresponding subgroups in G2.

Spearman correlation analysis was adopted to evaluate the correlation of contrast medium dose with CT values of liver parenchyma during HAP and PVP. In G1 and G2, the contrast medium dose was positively correlated with patient weight and CT values of liver parenchyma during HAP (all *P* values were <0.001). On the other hand, no clear correlation was identified between the contrast medium dose and the CT values of liver parenchyma during PVP (G1, *P* = 0.079; G2, *P* = 0.295) (Table 2).

**Figure 3:**
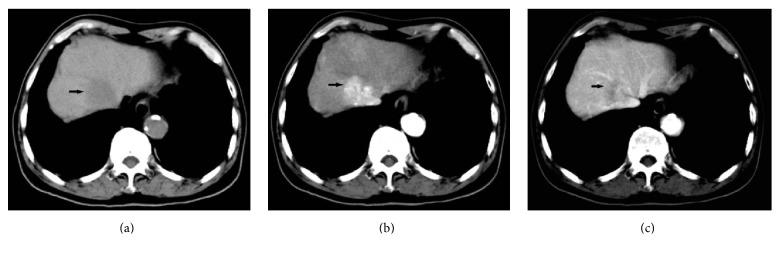
A 48-year-old male patient of G1 with HCC lesions was found liver occupied by ultrasound in physical examination. Unenhanced CT scans (a) revealed a liver S7 low-density round mass with clear boundary. Enhanced CT scans revealed significantly enhanced heterogeneous mass during HAP (b), the density of which was higher than the liver parenchyma of the same slice, and TLC value was 38 HU; the mass density in PVP (c) was lower than liver parenchyma and was enhanced during HAP and hypodense during PVP, and TLC value was −12 HU.

**Figure 4:**
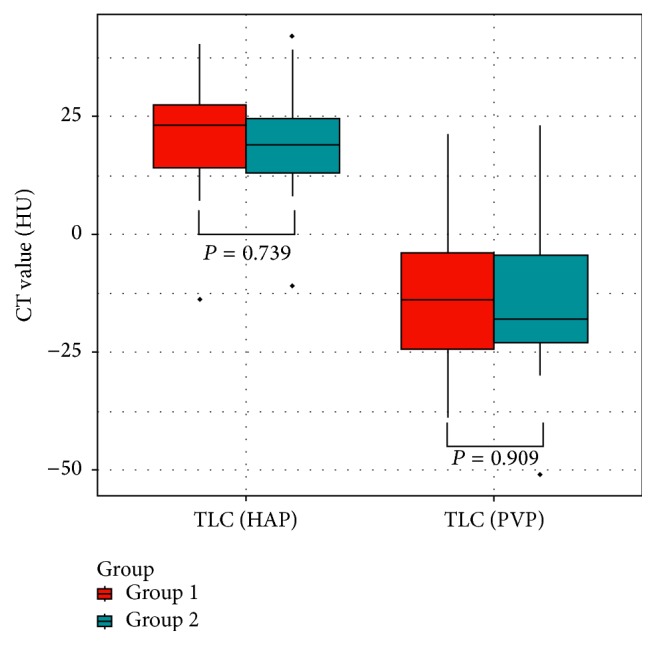
No statistical differences were found in the TLC values during HAP and during PVP between the two groups. The TLC values during HAP in G1 and G2 were 20.9 ± 11.8 HU and 19.5 ± 13.2 HU, respectively, while the TLC values during PVP in G1 and G2 were −14.7 ± 14.7 HU and −15.3 ± 16.8 HU, respectively.

A total of 31 lesions were detected in 23 patients with HCC in G1 with 3 being the highest number of lesions identified in one single patient. A total of 27 lesions were detected in the 15 patients with confirmed HCC in G2, with 4 being the highest number of lesions identified in one single patient. The TLC values during HAP in G1 and G2 were 20.9 ± 11.8 HU and 19.5 ± 13.2 HU, respectively, and −14.7 ± 14.7 HU and −15.3 ± 16.8 HU, respectively, during PVP (Figure 3). Between the two groups, there were no statistical significant differences demonstrated in the TLC values measured during HAP and PVP (Figure 4). The mean scores of tumor conspicuity of HCC lesions during HAP for G1 and G2 were 2.61 and 2.57, respectively, measured by one radiologist, and 2.47 and 2.53, respectively, measured by the other. The scores given during PVP for G1 and G2 were 2.13 and 2.17, respectively, by one radiologist and 2.33 and 2.17, respectively, by the other. The two radiologists were both in substantial conformity in grading the tumor conspicuity (G1: *κ* 0.693, *P* < 0.001; G2: *κ* 0.734, *P* < 0.001).

## 4. Discussion

Previous studies have evaluated the image quality of multiphase dynamic enhanced CT of liver using different contrast concentrations [21, 22]. It has been demonstrated by researchers that higher contrast concentrations resulted in better enhancement of liver, where lesions can be more easily identified. However, higher contrast concentration may result in higher osmotic pressure, which can increase the risk of adverse reactions such as contrast-induced nephrotoxicity [23]. Scholars have previously proposed and explored various protocols using fixed abdominal concentration by adjusting contrast medium dose based on body weight. Yamashita et al. [24] used the contrast medium dose of 1.5, 2.0, or 2.5 mL/kg or a fixed dose of 100 mL of iopamidol 300 to determine the optimal contrast dose for helical CT of the abdomen based on patient weight. They found the use of 2.0–2.5 mL/kg of intravenous contrast medium produced best results when compared to 1.5 mL/kg group and fixed dose group. Arana et al. [25] compared 1.75 ml/kg dosing regime and a fixed dose of 120 ml using the same nonionic contrast medium (320 mgI/ml) and found that an injection volume of 1.75 ml/kg offered a more optimal diagnostic quality. Compared to these previous studies, a lower the contrast medium dose was adapted for the injection protocol in this current study.

The individualized contrast injection protocol used in this study offers flexibility by providing the option of three dosing methods: weight factor, volume factor, and iodine load. The primary difference between these dosing methods lies in the variables (patient weight and concentration) used for calculating individualized contrast medium dose. Weight factor method was used in the present study, in which both patient weight and concentration were used to determine an individualized contrast medium dose. A fixed weight factor and concentration were set as default value for all patients; weight was the only variable required where the platform would then generate an individualized contrast medium injection protocol. Due to its easy-to-use characteristics, this unique optimized contrast medium dosing method provides individualized contrast medium injection protocol for each patient even under special clinical and research requirements.

It is well known that the liver uniquely receives a dual blood supply from the hepatic artery and the portal vein, which approximately contributes 25% and 75% of the total blood supply respectively. The arterial and portal blood mixes within the hepatic sinusoids before draining into the hepatic venous system [26]. Immediately after contrast medium injection, the contrast enhancement of the liver parenchyma is completely provided by the hepatic artery during the early HAP, and then the portal vein becomes involved in the late HAP and will be the major source of blood supply during PVP. Ichikawa et al. [27] reported that, during HAP, the amplitude of the contrast enhancement of well-arterial-perfused organs such as focal hypervascular hepatic lesions was dependent on the injection speed of contrast medium. Furthermore, the injection dose was one of the most important factors in determining the amplitude of the contrast enhancement of poorly arterial-perfused organs, such as the portal vein or liver parenchyma. Although the contrast medium injection dose of the two groups uses different weight-based protocols in this study, the contrast medium injection rate and contrast medium concentration remained fixed and therefore do not contribute to the differences in CT values of liver parenchyma during HAP. Hence, as the body weight increases, there would be a negative correlation between the contrast dose and the CT values of liver parenchyma during HAP in both groups. Moreover, George et al. [8] used 98 ml iodinated contrast medium (300 mgI/ml) delivered at 3 ml/s with the patient being scanned at 60 seconds, and they found statistically significant negative correlation between patient weight and radiodensity at the portal vein, aorta, spleen, and liver. This finding indicated that, as patient weight increases, the degree of enhancement will decrease during PVP when fixed contrast medium dosing regime was applied. However, in the current study, same fixed weight factor (0.4 mgI/kg) and volume factor (1.5 ml/kg) were applied to patients from both groups. Therefore, there was no negative correlation between the contrast dose and the CT values of liver parenchyma during PVP.

Moreover, the liver parenchyma ΔHU (CT value difference between plain and enhanced CT) during PVP decreased with higher body weight, especially in obese patients in this study. However, the liver parenchyma ΔHU during HAP of G1 and G2 did not demonstrate statistical significant differences. As most HCC are supplied by the hepatic arteries, the mass density of HCC lesions generally shows vivid enhancement during HAP. These lesions then become hypodense compared to the rest of the normal hepatic parenchyma while the blood was washed out during PVP. Guerrisi et al. [28] founded that, compared to an iodine concentration of 320 mgI/ml, contrast medium with an iodine concentration of 400 mgI/ml would significantly increase the conspicuity of HCC during HAP. Furthermore, Fujigai et al. [29] demonstrated hypervascular HCC could be better depicted during HAP with sufficient hepatic enhancement of 50 HU during delayed phase when a fixed concentration of 320 mgI/ml iodine with the injection plan of 630 mgI/kg was used instead of the contrast medium injection protocol of 525 mgI/kg. In this study, fixed injection speed and optimized contrast medium concentration were used. The scores of tumor conspicuity of HCC during HAP remained excellent in both groups. Although the contrast medium dose was lower in G1, there were no statistical differences in the CT values of normal liver parenchyma during HAP between the two groups. Furthermore, the TLC values and scores of tumor conspicuity of HCC were also comparable between the two groups. These findings suggest that individualized contrast medium injection protocol has the advantage of reducing contrast dose without significantly affecting the degree of enhancement of normal liver parenchyma and HCC lesions. The enhancement during PVP is also important for the diagnosis and differential diagnosis of liver diseases, especially for certain pathologies which are dependent on portal venous blood supply. As only the cases with normal liver or HCC were enrolled, whether the decrease in the degree of hepatic enhancement during PVP could impact on the diagnosis and differential diagnosis for liver diseases other than HCC remains uncertain. In order to avoid these possible limitations, we suggest incremental increase to the weight factor for obese patients to maintain a steady enhancement effect. However, further studies are required to identify the optimal method in adjusting weight factor safely and efficiently for this group of patients.

There are limitations to the study. Firstly, although body weight is the most important factor affecting the degree of contrast enhancement in liver, others such as heart rate and vascular conditions are also important factors. Therefore, multiple linear regression models should be used in future studies to establish the correlation of CT values of liver parenchyma in dual phases with the contrast medium dose and cardiac output. This will help to evaluate and predict the function and influence of various factors on the contrast enhancement of liver after adjusted contrast medium doses based on body weight are applied. Secondly, patients with diffuse liver disease such as cirrhosis were excluded out in our study. Liver cirrhosis can influence haemodynamics of the liver where the enhancement of the hepatic parenchyma in dynamic CT is different from the normal liver, and the use of individualized contrast medium injection protocol in patient liver cirrhosis becomes difficult to evaluate. Therefore, patients with liver cirrhosis were excluded from this study to achieve good repeatability and controllability of results. Further studies are required to explore the optimal individual contrast medium injection protocol to improve the imaging quality with reasonable contrast medium dose in patients with liver cirrhosis.

## 5. Conclusion

In enhanced CT scan for liver imaging, individualizing the contrast dose based on the patient weight via contrast medium injection protocol can effectively reduce contrast medium dose without affecting the image quality.

## Figures and Tables

**Table 1:** Results of CT values of liver parenchyma and liver parenchyma ΔHU during HAP and PVP, CT values of portal vein during PVP, and contrast medium dose in G1 and G2.

	CT values of liver parenchyma during HAP (HU)	CT values of liver parenchyma during PVP (HU)	CT values of portal vein during PVP (HU)	Liver parenchyma ΔHU during HAP (HU)	Liver parenchyma ΔHU during PVP (HU)	Contrast medium dose (ml)
G1	77.3 ± 11.9	102.6 ± 9.5	147.0 ± 15.4	21.1 ± 11.0	46.4 ± 9.5	78.2 ± 12.8
G2	76.0 ± 11.5	106.4 ± 11.3	159.7 ± 18.4	18.5 ± 10.7	49.0 ± 10.2	93.0 ± 15.0
*P*	0.367	0.001	<0.001	0.059	0.021	<0.001

**Table 2:** Analysis on the correlation of contrast medium dose with body weight and CT values of liver parenchyma during HAP and PVP in G1 and G2.

		Body weight	CT values of liver parenchyma during HAP	CT values of liver parenchyma during PVP
Contrast medium dose for G1	*r* value	0.974	−0.517	−0.119
	*P* value	<0.001	<0.001	0.079
Contrast medium dose for G2	*r* value	0.983	−0.406	−0.11
	*P* value	<0.001	<0.001	0.295

## Abbreviations

CIN: Contrast-induced nephropathy
CT: Computed tomography
HAP: Hepatic arterial phase
HCC: Hepatocellular carcinoma
HIPAA: Health Insurance Portability and Accountability Act
ICM: Iodinated contrast medium
IRB: Institutional Review Board
PVP: Portal venous phase
ROI: Regions of interest
TLC: Tumor-liver contrast.
